# Occurrence and Concentrations of Toxic VOCs in the Ambient Air of Gumi, an Electronics-Industrial City in Korea

**DOI:** 10.3390/s150819102

**Published:** 2015-08-05

**Authors:** Sung-Ok Baek, Lakshmi Narayana Suvarapu, Young-Kyo Seo

**Affiliations:** Department of Environmental Engineering, Yeungnam University, Gyeongsan 712-749, Korea; E-Mails: suvarapu@gmail.com (L.N.S.); youngkyo@ynu.ac.kr (Y.-K.S.)

**Keywords:** VOCs, BTEX, HAPs, ambient air, electronics industry, Gumi city

## Abstract

This study was carried out to characterize the occurrence and concentrations of a variety of volatile organic compounds (VOCs) including aliphatic, aromatic, halogenated, nitrogenous, and carbonyl compounds, in the ambient air of Gumi City, where a large number of electronics industries are found. Two field monitoring campaigns were conducted for a one year period in 2003/2004 and 2010/2011 at several sampling sites in the city, representing industrial, residential and commercial areas. More than 80 individual compounds were determined in this study, and important compounds were then identified according to their abundance, ubiquity and toxicity. The monitoring data revealed toluene, trichloroethylene and acetaldehyde to be the most significant air toxics in the city, and their major sources were mainly industrial activities. On the other hand, there was no clear evidence of an industrial impact on the concentrations of benzene and formaldehyde in the ambient air of the city. Overall, seasonal variations were not as distinct as locational variations in the VOCs concentrations, whereas the within-day variations showed a typical pattern of urban air pollution, *i.e.*, increase in the morning, decrease in the afternoon, and an increase again in the evening. Considerable decreases in the concentrations of VOCs from 2003 to 2011 were observed. The reductions in the ambient concentrations were confirmed further by the Korean PRTR data in industrial emissions within the city. Significant decreases in the concentrations of benzene and acetaldehyde were also noted, whereas formaldehyde appeared to be almost constant between the both campaigns. The decreased trends in the ambient levels were attributed not only to the stricter regulations for VOCs in Korea, but also to the voluntary agreement of major companies to reduce the use of organic solvents. In addition, a site planning project for an eco-friendly industrial complex is believed to play a contributory role in improving the air quality of the city.

## 1. Introduction

Over the past two decades there has been a rapid increase in urbanization and industrialization in Korea. With this has come a dramatic increase in the number of manufacturing facilities, residences, and office buildings, together with increases in both the number and density of motor vehicles. As the total area of South Korea is very small, most urban areas are densely populated and new towns have developed rapidly in the vicinity of industrial complexes. Thus, industrial emissions and motor vehicles are believed to be the major causes of ambient air pollution in most Korean cities [1].

Recently, emissions of hazardous air pollutants (HAPs), particularly toxic volatile organic compounds (VOCs), from large industrial complexes have been of great concern in Korea [2,3]. Gumi City is a typical industrial city in Korea with a population of approximately 450,000. The Gumi National Industrial Complexes (GNIC) is often called the Korean Silicon Valley and the Mecca of the Korean electronics industry. GNIC plays a key role in the South Korean economy, being responsible for a major proportion of the country’s exports. More than 1700 companies with approximately 90,000 workers are located in the GNIC. Among the industries in the city, 84% are electronic companies, 6% are petro-chemical, 4% are non-metal, 3% are textile and 3% are other industries [4]. The electronic industry is occasionally regarded as an “environment-friendly” or “stack-free” business because it consumes very little fossil fuels and industrial water. However, in general, the electronic industries use a variety of organic and/or inorganic solvents in their manufacturing processes [1]. As a result, the fugitive emissions of toxic chemicals volatilized from the industrial processes may cause unknown air quality problems in such a large industrial city [5]. Therefore, monitoring of the ambient air quality with respect to toxic VOCs is an important task in view of the health of workers and residents living around the industries [6].

Although questions have been raised from the general public and NGOs in Gumi City regarding the quality of ambient air [5,7], the air quality data that is available for this city has not been adequate to provide accurate information on the real-life situation to policy makers. This is largely because the air quality data available in the city focuses mostly on criteria pollutants, such as particulate matter, sulfur dioxide and nitrogen oxides, whereas little attention has been given to non-criteria pollutants, such as VOCs. In Korea, the first and only ambient air quality standard associated with the VOCs appeared in 2007 for benzene, which is 1.5 ppb as an annual average [1]. Before developing a solid scheme for the effective management of air quality problems, scientific information on at least three factors should be provided, which are abundance, ubiquity and toxicity of a wide range of target pollutants.

Environmental implications of VOCs can be summarized into two aspects, *i.e.*, one is that many VOCs (particularly aliphatic hydrocarbons such as olefins) play an important role as the precursors of secondary air pollutants, such as ozone, aldehydes and organic aerosols [8], and the other is that some VOCs (particularly aromatics and halogenated ones) can have adverse effects on human health [8]. As an example, benzene is considered one of the most important VOCs because of its carcinogenicity. Benzene and formaldehyde are classified as group 1 carcinogens (proven carcinogen to humans) by the International Agency for Research on Cancer [9]. Exposure to other VOCs, such as toluene, ethylbenzene and trichloroethylene, may also have adverse health effects on humans [10].

High levels of ozone in the summertime are now very common phenomena everywhere in Korea, due to the VOCs and nitrogen dioxide from vehicle emissions and industrial sources [1]. On the other hand, the ozone problem in Gumi City has been less severe than other urban and industrial areas in Korea [11]. Therefore, if there is an air quality problem associated with VOCs in the city, the nature of the problem can be inferred to be related to health implications rather than the ozone issues, because a large amount of organic solvents are used in this city. According to the Korea Pollutant Release and Transfer Registers (PRTR) Data for 2009 [12], the top five VOCs released into the air of the city are toluene (65.4 ton/year), trichloroethylene (53.8 ton/year), xylenes (37.3 ton/year), methyl ethyl ketone (23.0 ton/year), and tetrachloroethylene (21.9 ton/year), comprising more than 99% of the VOCs emissions identified from industrial sources within the city.

Over the past few decades, a considerable number of studies have been conducted on atmospheric VOCs in urban and industrial areas [13,14,15,16,17,18,19,20,21,22,23,24,25,26]. Most studies focused on vehicle emissions in large urban areas [13,14,15,16,17] or the formation of secondary air pollutants as photochemical byproducts [18,19]. Regarding industrial VOCs, several field studies have been reported for different types of industries, such as petrochemical, oil refinery, chemical processing, steel industries, and painting processes [20,21,22,23,24,25,26,27]. On the other hand, little is known about the occurrence and concentrations of VOCs associated with the electronic industry.

The main aim of this study was to characterize the occurrence and concentrations of a wide range of VOCs including aliphatic, aromatic, nitrogenous, halogenated, and carbonyl compounds in the ambient air of Gumi City. The temporal and spatial variations of ambient levels of VOCs were investigated by field monitoring in several sites in the city covering four seasons from 2003 to 2004 (first campaign), and from 2010 to 2011 (second campaign). More specific objectives of this study were: (i) to provide quantitative information on the concentrations of toxic VOCs in residential, commercial and industrial areas in the city; (ii) to investigate the extent to which industrial sources influence the air quality in non-industrial areas in the city; and (iii) to assess the impact of environmental policies on the reduction of VOCs emissions by investigating the long term trends of the VOCs concentrations in the city.

## 2. Experimental Section

### 2.1. Description of Sampling Sites

**Figure 1:**
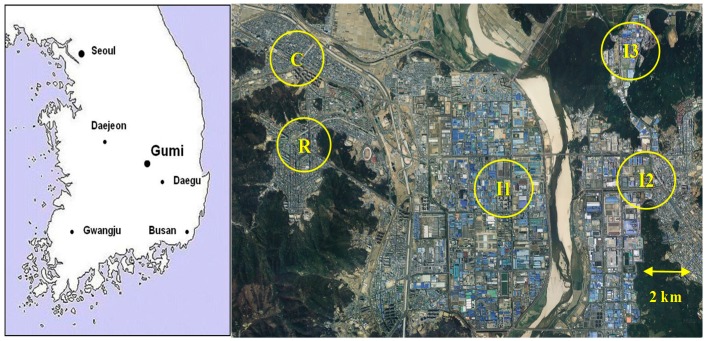
Locations of Gumi City in Korea and sampling sites in the city: I1—industrial site I, I2—industrial site II, I3—industrial site III; R—residential site; C—commercial site.

To characterize the atmospheric VOCs in Gumi City, two field campaigns were carried out at 7 year intervals, *i.e.*, the first was from December 2003 to November 2004 and the second was from April 2010 to March 2011. In the first campaign, ambient air samples were collected at five sites in the city, *i.e.*, three industrial, one residential and one commercial site, as shown in Figure 1. Industrial site I was located in the center of the oldest and the largest complex; thus particular attention was given to this site. Industrial site II was in a mixed zone of half industrial and half commercial/residential areas. Nevertheless, it was named as an industrial site because big companies, such as LG and Samsung, are located near the sampling site; hence considerable impacts of industrial sources are expected. Industrial site III was located within the smallest complex in a valley, which was isolated geographically from the other complexes. This site was excluded for the second campaign because many companies in this complex had moved to a new complex developed in the outskirts of the city (information on the locations of the GNIC can be found in a website [4]). Instead, the frequency of sampling at other sites was increased during the second campaign. The residential site was located in a new town, being surrounded by a large number of apartment buildings. Finally, the commercial site was in a traditional old town, where many shopping centers and office buildings were concentrated. All sampling sites were well prepared for air sampling purposes in terms of electricity and vandalism, because these sites all belong to the National Air Quality Monitoring Network in Korea.

### 2.2. Sampling Period, Frequency and Duration

The sampling periods, frequencies and duration for the two campaigns are summarized in Table 1. For both campaigns, air sampling was conducted in four seasons at each site, *i.e.*, spring, summer, fall, and winter. In each season, sampling was carried out over seven consecutive days in each site. One exception is industrial site I, where samples were taken every six days throughout the year for the first campaign (but not in the second campaign). This site was identified as the most important site, as mentioned earlier.

During the first campaign, all the VOCs samples were collected manually in duplicate, and the average of the two measurements was regarded as one data-point. Each sampling was carried out for 150 min and three times per day, *i.e.*, morning (09:00~11:30), afternoon (13:00~15:30), and evening (18:30~21:00). On the other hand, single samples were collected automatically during the second campaign, and the sampling frequency was increased to six times per day (00:00~04:00, 04:00~08:00, and so on). For carbonyl compounds, the sampling frequency and duration were same as the VOC samples for the first campaign, while only two samples per day were collected for the second campaign, *i.e.*, one in the morning (09:00~11:00) and the other in the afternoon (13:00~15:00). As a result, 507 and 668 samples for VOCs (476 and 212 for carbonyls) were collected during the first and second campaigns, respectively.

**Table 1:** Sampling sites and periods for the first and the second campaigns.

	2003/2004 Campaign	2010/2011 Campaign
Sampling site *	3 industrial sites (I, II & III)	2 industrial sites (I & II)
	1 residential site	1 residential site
	1 commercial site	1 commercial site
Sampling period **	Winter: 13~19 January 2004	Spring: 21~27 May 2010
	Spring: 9~15 April 2004	Summer: 3~9 August 2010
	Summer: 13~19 August 2004	Fall: 15~21 October 2010
	Fall: 15~21 October 2004	Winter: 5~11 January 2011
Sampling method	Manual sampling	Automatic sampling
Sampling flowrate	150 mL/min for VOCs	100 mL/min for VOCs
	1 L/min for carbonyls	1 L/min for carbonyls
Sampling duration	2.5 h (150 min) for VOCs	4 h (240 min) for VOCs
	2.5 h (150 min) for carbonyls	2 h (120 min) for carbonyls
Sampling frequency	3 per day for VOCs and carbonyls	6 per day for VOCs and 2 per day for carbonyls
Number of samples	507 for VOCs and 476 for carbonyls	668 for VOCs and 212 for carbonyls

* In the first campaign, industrial site I was selected as a supersite, and industrial site III was excluded in the second campaign. ** In the first campaign, sampling was carried out every 6 days at industrial site I from December 2003 to November 2004.

### 2.3. Sampling and Analytical Methods

The protocol of the VOCs measurement methods used in this study was in principle similar to the USEPA TO-17 [28]. For the 2010–2011 campaign, the VOC samples were collected at a flow rate of 100 mL/min on stainless steel adsorbent tubes (1/4″ × 9 cm), using low flow rate pumps equipped with a mass flow controller (Flec pump, Chematec, Roskilde, Denmark) and sequential automatic tube samplers (STS 25, Perkin Elmer, Beaconsfield, UK and MTS32, Markes, Llantrisant, UK). Each adsorbent tube was packed with 120 mg of Carbograph 2 (20/40 mesh) in the front position and 280 mg of Carbograph 1 (40/60 mesh) in the back position. Before sampling, the adsorbent tubes were preconditioned with helium as a carrier gas at 250 °C for 2 h using a thermal conditioner (TC-20, Markes). The preconditioned tube was kept in a 50 mL vial with a PTFE lined cap. The analysis of VOCs was performed using a GC/MS (HP6890/5973, Hewlett-Packard, Wilmington, DE, USA) with an automatic thermal desorption apparatus (Unity/Ultra, Markes). During the thermal desorption process, the eluted VOCs from the sampling tube were transferred to a cold trap (packed with 12 mg of Tenax TA and 47 mg of Carbotrap) at 300 °C and a flow rate of 50 mL/min for 10 min. Subsequently, the cold trap was heated rapidly from −15 °C to 320 °C, and maintained at that temperature for 5 min. The VOCs were then injected onto a capillary column (Rtx-1, 0.32 mm × 105 m × 1.5 μm, Restek, Bellefonte, PA, USA). The initial temperature of the GC oven was set to 50 °C for 10 min, then increased to 250 °C at a rate of 5 °C/min. The valves of the thermal desorber and transfer line were maintained at 180 °C. The carrier gas (helium) flow rate in the column was 1.4 mL/min (15 psi) and the outlet split flow of the thermal desorber was 10 mL/min.

For the 2003–2004 campaign, the VOCs were collected by drawing air through a stainless steel sampling tube (1/4″ × 9 cm) containing 100 mg of Carbotrap-C (equivalent to Carbograph 2) backed up with 300 mg of Carbotrap (equivalent to Carbograph 1) at a flow rate of 150 mL/min. The analysis of VOCs was performed using an automatic thermal desorption unit (ATD-400, Perkin Elmer) connected to the GC/MS. The GC column and operating conditions of the analytical system for the first campaign were virtually the same as those for the second campaign.

For both campaigns, carbonyl compounds were collected on DNPH-silica cartridges (LpDNPH S10L, Supelco, Bellefonte, PA, USA) at a flow rate of 1 L/min. An ozone scrubber (Supelco) was placed in front of the DNPH cartridge. The carbonyls were extracted with 3 mL of acetonitrile, and then analyzed by HPLC with UV detection at 360 nm (Shimadzu HPLC system, Kyoto, Japan). Details of the carbonyl sampling and analytical methods used in this study can be found elsewhere [7,29].

### 2.4. Target Analytes and Preparation of Standard Samples

In this study, the target VOCs for the two campaigns were slightly different due to a difference in the standard mixtures used for calibration. During the first campaign, two types of gas standards were used, *i.e.*, a TO-14A 41 Component Mix (1 ppm, Restek, Bellefonte, PA, USA) and a mixture of 57 ozone precursors (1 ppm, Restek). Some of VOCs were duplicated in the two mixtures and some very volatile VOCs, such as C_2_~C_3_ hydrocarbons could not be measured using the sampling and analytical methods of this study. As a result, a total of 83 individual VOCs were finally selected as the target analytes. For the second campaign, an EPA TO-15/17 Calibration Mix (1 ppm, Supelco) containing 62 components were used. Among the 62 compounds, four very volatile VOCs (propylene, ethanol, Freon 12, and chloromethane) were excluded from the determination because these compounds were found to be collected inefficiently by the adsorbent tubes used in this study. Methyl ethyl ketone (MEK) was included in the 62 Mix, but this compound was determined by HPLC to be a carbonyl compound. Acrylonitrile was not included in the 62 Mix, but this compound was calibrated with the 41 Mix standards. In addition, for some important VOCs, which were unavailable in the gas standard mixtures, a liquid standard mixture was prepared with individual liquid standards (Sigma-Aldrich Co., St. Louis, MO, USA). These were naphthalene, *N*,*N*-dimethylformamide (DMF), epichlorohydrin, nitrobenzene, aniline, phenol, 2-methoxyethanol, 2-ethoxyethaol, and 2-ethoxyethylacetate. These 9 VOCs are of concern in Korea, as being included among 48 priority air toxics [3]. As a result, a total of 67 compounds were determined for the second campaign. A self-manufactured spiking apparatus [30,31] was used to prepare the standard samples by spiking known amounts of the gas standard mixture into pre-conditioned tubes. The liquid standard samples were prepared by spiking the standard into cleaned adsorbent tubes using a packed column injector ofthe GC at 250 °C and a helium flow rate of 100 mL/min [32]. In addition to these compounds, 15 carbonyl compounds were determined for both campaigns using a standard mixture (TO11/IP-6A Aldehyde/Ketone-DNPH Mix., Supleco) [33].

### 2.5. Quality Control and Quality Assurance

An evaluation of duplicate precision for the VOC samples analyzed by the adsorption and thermal desorption method is essential because replicate analysis is practically impossible for such samples. According to the TO-17, a criterion of 30% for duplicate precision was recommended for the sorbent-based sampling of VOCs. In this study, the mean duplicate precision (MDP) was within 30% for the majority of the target VOCs, and the within-a-day repeatability appeared to be less than 10% overall, while the between-days repeatability was less than 20%. The method detection limits (MDLs) for each target VOC were evaluated according to the USEPA guidelines [34], and the MDLs were estimated to be 0.01~0.05 ppb, depending on the individual compounds. More details on the performance of the VOC sampling and analytical methods used in this study can be found elsewhere [30,31,32,33]. Until now, no standard reference materials (SRM) are available for VOC samples. In this study, as an alternative approach to evaluate the accuracy of VOC measurements, inter-lab comparison studies were carried out with a “third party” laboratory at the Korea Research Institute of Standards and Science (KRISS) by sharing parts of duplicate samples (*n* = 26) [5]. The results of the inter-lab comparisons showed that the MDPs for toluene and trichloroethylene were 27.5% and 19.2%, respectively, while benzene was 32.1%. More volatile compounds, such as dichloromethane, appeared to be less accurate (50.7%). However, most of target VOCs showed a MDP of 30% or lower.

## 3. Results and Discussion

In this study, a very wide range of VOCs were determined. The monitoring data will be discussed with respect to their frequencies of detection, locational, and temporal variations. A particular emphasis will be given on the comparison of concentrations in “industrial” and “non-industrial” areas to evaluate industrial impacts on the ambient levels of VOCs in Gumi city. Finally, data from the two campaigns will be compared to investigate a long term trend in the VOC concentrations in the city.

### 3.1. Occurrence of VOCs in the Ambient Air of Gumi City

In Gumi City, the most ubiquitous VOCs in ambient air appeared to be benzene, toluene, *m,p*-xylenes, formaldehyde, acetaldehyde, and MEK because they have been detected in every sample during both campaigns. The frequencies of detection for individual compounds are summarized in Table 2. In this study, the detection frequency is defined as the percentage of the number of samples over the MDL for the total number of effective samples. For the first campaign, 32 compounds among 98 target compounds were detected in a more than 50% frequency, while 24 compounds have not been detected in any sample. During the second campaign, the detection frequencies of 26 among 82 target analytes were more than 50%, while 39 compounds were detected in less than 5% of the total samples. In this paper, therefore, the discussion will focus on a number of selected VOCs with respect to their abundance, ubiquity and toxicity.

**Table 2:** Frequencies of detection for the target VOCs in the ambient air of Gumi city.

Detection Frequency	First Campaign (2003/2004)	Second Campaign (2010/2011)	
100%~95%	benzene, toluene, *m,p*-xylene, pentane, 2-methylpentane, formaldehyde, acetaldehyde, acetone, methyl ethyl ketone	benzene, toluene, ethylbenzene, *m,p*-xylenes, hexane, vinylacetate, methyl tert-butyl ether, *o*-xylene, formaldehyde, acetaldehyde, acetone, methy ethyl ketone	
75%~95%	isopentane, ethylbenzene, isobutane, hexane, dichloromethane, methylcyclopentane, propionaldehyde, trichloroethylene	ethyl acetate, carbon tetrachloride, heptane, 1,2,4-trimethylbenzene, methyl isobutyl ketone, cyclohexane, propionaldehyde	
50%~75%	3-methylpentane, butane, o-xylene, 1-butene, heptane, decane, nonane, dodecane, 3-methyl- hexane, m,p-ethyltoluene, 1,2,4-trimethylbenzene, crotonaldehyde, iso-valeraldehyde	naphthalene, trichloroethylene, styrene, 1,3,5-trimethylbenzene, crotonaldehyde, iso-valeraldehyde		
25%~50%	2-methylhexane, *n*-octane, 2,3-dimethylbutane, methylcyclohexane, styrene, Freon 11, cyclohexane, butyraldehyde	Freon 113, 2-propanol, phenol, 4-ethyltoluene, 1,2-dichloropropane, Freon 11, tetrachloroethylene, 1,1,1-trichloroethane, *N*,*N*-dimethylformamide	
5%~25%	tetrachloroethylene, 1-pentene, isoprene, 2-pentene, *trans*-2-butene, cyclopentane, 3-methylheptane, 1,1,1-trichloroethane, *cis*-2-pentene, 2-methyl- heptane, 1,3,5-trimethylbenzene, 1,2,3-trimethyl benzene, 1,2-dichloropropane, 2,2,4-trimethyl pentane, *o*-ethyltoluene, 2,3-dimethylpentane, *p*-diethylbenzene, carbon tetrachloride, 2,4-dimethylpentane	1,2-dichloroethane, chloroform, 2-methoxyethanol, dichloromethane, tetrahydrofuran, 1,4-dioxane, carbon disulfide, butyraldehyde	
0%~5%	*cis*-2-butene, 2,3,4-trimethylpentane, chloroform, *n*-propylbenzene, 1,3-butadiene, 2,2-dimethyl- butane, Freon 12, 1-hexene, Freon 113, 1,4-dichlorobenzene, chlorobenzene, 1,2,4-trichloro- benzene, chloroethane, isopropylbenzene, 1,2-dichlorobenzene, acrylonitrile, 1,1,2,2-tetra- chloroethane, 1,1-dichloroethene, 1,2-dichloro- ethane, Freon 114, vinyl chloride, bromomethane, 1,1-dichloroethane, acrolein, *cis*-1,2-dichloro-ethylene, 1,2-dibromoethane, *trans*-1,3-dichloro-propene, 1,1,2-trichloroethane, *cis*-1,3-dichloro-propene, 1,3-dichlorobenzene, *m*-diethylbenzene, bromodichloromethane, bromoform, dibromo-chloromethane, *trans*-1,2-dichloroethylene, valeraledehyde, 1,2,3-trichlorobenzene, hexaaldehyde, *o,m,p*-tolualdehyde, 2,5-dimethyl-benzaldehyde	2-ethoxyethanol, 1,3-butadiene, chlorobenzene, acrylonitrile, Freon 114, 1,1-dichloroethene, 1,2-dichlorobenzene, *trans*-1,2-dichloro-ethylene, Freon 12, 2-ethoxyethylacetate, benzyl chloride, 1,2,4-trichlorobenzene, 2-hexanone, bromomethane, 1,1-dichloroethane, bromodichloromethane, 1,1,2,2-tetrachloro- ethane, 1,3-dichlorobenzene, 1,4-dichloro-benzene, vinyl chloride, chloroethane, bromoform, *cis*-1,2-dichloroethylene, *cis*-1,3-dichloropropene, *trans*-1,3-dichloro-propene, 1,1,2-trichloroethane, dibromo- chloromethane, 1,2-dibromoethane, hexachloro-1,3-butadiene, anilin, epichlorohydrin, nitrobenzene, acrolein, *n*-valeraldehyde, hexaaldehyde, *o,m,p*-tolualdehyde, 2,5-dimethyl- benzaldehyde	

### 3.2. Spatial Variations of VOCs Concentrations

In order to investigate locational distributions of VOCs within Gumi City, the concentration data at each sites for the selected VOCs from the first and the second campaigns are summarized in Table 3 and Table 4, respectively. A wide range of concentrations were documented. For example, the toluene and trichloroethylene levels during the first campaign ranged from 0.40 ppb to 50.78 ppb, and from “less than MDL” to 38.11 ppb, respectively. The highest concentration of 77.71 ppb was observed for acetaldehyde in the first industrial site of the first campaign. On the other hand, the ambient levels and variations of VOCs during the second campaign appeared to be considerably lower than in the first campaign, indicating that there have been many changes during the seven year period not only within the city, but in the governmental policies for improving the air quality in Korea. This issue will be discussed more intensively in the later part of this paper.

For the first campaign, toluene appeared to be the most abundant (5.60 ppb as a mean of total data, *n* = 507) among the VOCs group, followed in order by *n*-pentane (2.19 ppb), trichloroethylene (1.34 ppb) and *m,p*-xylenes (0.88 ppb). Among the carbonyl group, acetaldehyde (4.44 ppb, n = 476) was the most abundant compound, followed by formaldehyde (3.55 ppb), acetone (3.31 ppb) and MEK (2.74 ppb). Most of these compounds are strongly associated with industrial manufacturing processes, while formaldehyde in the urban atmosphere is commonly derived from both indoors and outdoors [35]. MEK, another well-known organic solvent used in industrial settings, is also emitted as a combustion product from motor vehicles, landfills and so forth [36].

**Table 3:** Concentrations (in ppb) of selected VOCs in Gumi city during 2003/2004.

VOCs	Industrial Site I (*n* = 179)		Industrial Site II (*n* = 82)		Industrial Site III (*n* = 82)		Residential Site (*n* = 82)		Commercial Site (*n* = 82)	
	Mean ± SD ^1)^	Max ^2)^	Mean ± SD	Max	Mean ± SD	Max	Mean ± SD	Max	Mean ± SD	Max
*n*-Butane	0.39 ± 0.44	2.21	0.40 ± 0.56	2.93	0.68 ± 0.76	3.28	0.24 ± 0.28	1.87	0.30 ± 0.32	1.49
iso-Pentane	0.78 ± 0.67	4.51	0.62 ± 0.52	1.99	1.67 ± 1.30	7.79	0.47 ± 0.35	1.63	0.55 ± 0.38	2.14
*n*-Pentane	1.90 ± 2.51	26.16	1.09 ± 1.23	6.71	7.39 ± 7.86	38.99	0.45 ± 0.38	1.74	0.43 ± 0.30	1.41
Dichloromethane	1.24 ± 1.27	8.16	0.63 ± 0.68	3.47	0.31 ± 0.30	1.28	0.55 ± 0.59	3.82	0.72 ± 0.65	3.88
2-Methylpentane	0.81 ± 0.73	6.10	0.59 ± 0.49	2.83	0.82 ± 0.51	4.16	0.43 ± 0.27	1.41	0.52 ± 0.31	1.36
3-Methylpentane	0.34 ± 0.39	3.27	0.25 ± 0.22	0.97	0.42 ± 0.40	2.52	0.17 ± 0.16	0.90	0.22 ± 0.19	1.10
n-Hexane	0.53 ± 0.62	5.92	0.53 ± 0.49	2.17	1.21 ± 1.31	8.49	0.36 ± 0.20	0.93	0.39 ± 0.32	2.29
Benzene	0.54 ± 0.28	1.64	0.68 ± 0.33	1.88	0.69 ± 0.31	1.91	0.60 ± 0.27	1.65	0.67 ± 0.33	1.71
Carbon tetrachloride	0.07 ± 0.03	0.20	0.08 ± 0.02	0.13	0.05 ± 0.01	0.09	0.08 ± 0.02	0.13	0.06 ± 0.02	0.11
Trichloroethylene	3.12 ± 5.29	38.11	0.49 ± 0.47	2.04	0.47 ± 0.65	3.60	0.20 ± 0.25	1.37	0.31 ± 0.49	2.57
Toluene	5.50 ± 5.12	37.12	7.93 ± 8.69	50.78	8.95 ± 6.32	32.05	2.82 ± 1.96	9.54	2.43 ± 1.70	8.39
Tetrachloroethylene	0.13 ± 0.35	3.89	0.08 ± 0.06	0.37	0.24 ± 0.28	1.75	0.04 ± 0.04	0.30	0.05 ± 0.03	0.22
Ethylbenzene	0.54 ± 0.66	5.77	0.54 ± 0.70	5.33	0.50 ± 0.32	1.93	0.28 ± 0.21	1.77	0.34 ± 0.35	2.55
*m,p*-Xylenes	1.10 ± 1.29	9.17	0.96 ± 0.97	5.99	1.00 ± 0.58	2.51	0.57 ± 0.55	2.91	0.48 ± 0.34	1.98
Styrene	0.23 ± 0.34	3.30	0.10 ± 0.07	0.35	0.31 ± 0.36	1.59	0.05 ± 0.04	0.40	0.05 ± 0.06	0.39
*o*-Xylene	0.30 ± 0.33	2.21	0.28 ± 0.29	1.84	0.31 ± 0.19	0.76	0.16 ± 0.15	0.72	0.14 ± 0.10	0.63
1,2,4-TMB ^3)^	0.18 ± 0.21	1.39	0.20 ± 0.20	1.06	0.31 ± 0.24	1.08	0.11 ± 0.10	0.49	0.13 ± 0.11	0.56
*n*-Decane	0.43 ± 0.48	3.25	0.31 ± 0.54	4.20	1.91 ± 1.90	8.85	0.14 ± 0.15	0.80	0.18 ± 0.21	1.39
*n*-Nonane	0.25 ± 0.41	3.22	0.22 ± 0.37	2.56	1.49 ± 2.24	16.60	0.11 ± 0.11	0.62	0.15 ± 0.16	0.90
Formaldehyde *	3.40 ± 1.66	9.10	3.86 ± 2.01	12.45	3.87 ± 1.90	10.31	3.46 ± 1.77	11.10	3.36 ± 1.62	7.85
Acetaldehyde *	7.35 ± 9.19	77.71	4.22 ± 3.22	15.69	2.52 ± 1.90	9.72	2.34 ± 1.21	5.43	2.05 ± 0.95	5.45
Acetone *	3.28 ± 1.56	11.06	3.59 ± 1.49	9.07	4.27 ± 2.70	15.61	2.74 ± 1.36	7.45	2.68 ± 1.63	13.38
MEK * ^4)^	2.40 ± 1.97	12.98	3.32 ± 2.64	13.28	4.81 ± 4.74	28.13	1.80 ± 1.12	4.79	1.71 ± 1.25	6.75
Propionaldehyde *	0.80 ± 0.90	4.11	1.04 ± 1.36	7.25	1.01 ± 1.18	6.19	1.21 ± 1.39	5.33	1.03 ± 1.12	5.61
Crotonaldehyde *	0.31 ± 0.31	2.06	0.36 ± 0.44	1.85	0.61 ± 0.69	4.12	0.10 ± 0.12	0.65	0.17 ± 0.30	2.09

^1)^ Standard deviation; ^2)^ Maximum; ^3)^ 1,2,4-Trimethylbenzene; ^4)^ Methyl ethyl ketone. * The number of samples for the carbonyl compounds are 172, 78, 77, 75, and 74 for the industrial sites #1, #2, #3, residential, and commercial site, respectively.

Overall, the most abundant VOC for the second campaign was also toluene (2.06 ppb, *n* = 668), followed by ethyl acetate (0.65 ppb), *m,p*-xylenes (0.38 ppb) and benzene (0.35 ppb). For carbonyl compounds, the mean concentration (2.21 ppb, *n* = 212) of acetaldehyde decreased to a half level of the first campaign, while formaldehyde (3.46 ppb) almost the same as before. On the other hand, such comparison of the rankings for the two campaigns may not be appropriate because of the difference in the target compounds for each campaign.

**Table 4:** Concentrations (in ppb) of selected VOCs in Gumi City during 2010/2011.

VOCs	Industrial Site I (*n* = 168)		Industrial Site II (*n* = 168)		Residential Site (*n* = 168)		Commercial Site (*n* = 164)	
	Mean ± SD ^1^^)^	Max ^2^^)^	Mean ± SD	Max	Mean ± SD	Max	Mean ± SD	Max
Isoproyl alcohol	0.71 ± 1.91	18.84	0.23 ± 0.52	3.62	0.03 ± 0.08	0.58	0.06 ± 0.26	2.14
Dichloromethane	0.03 ± 0.12	0.88	0.09 ± 0.40	3.51	0.01 ± 0.04	0.29	0.05 ± 0.26	2.18
MTBE ^3^^)^	0.18 ± 0.14	0.66	0.25 ± 0.26	1.22	0.25 ± 0.31	2.21	0.26 ± 0.27	1.54
Ethyl acetate	1.25 ± 1.71	10.30	0.79 ± 1.20	8.61	0.24 ± 0.32	2.09	0.30 ± 0.80	6.94
n-Hexane	0.23 ± 0.15	0.97	0.26 ± 0.19	1.42	0.26 ± 0.38	4.01	0.24 ± 0.22	1.24
Vinyl acetate	0.31 ± 1.02	2.09	0.29 ± 1.22	2.26	0.23 ± 0.75	1.31	0.24 ± 0.52	1.01
Benzene	0.32 ± 0.18	0.81	0.34 ± 0.21	0.98	0.32 ± 0.21	0.97	0.41 ± 0.46	2.75
Carbon tetrachloride	0.07 ± 0.17	0.26	0.06 ± 0.10	0.16	0.07 ± 0.05	0.12	0.07 ± 0.07	0.19
Trichloroethylene	0.41 ± 0.46	2.70	0.27 ± 0.33	1.83	0.06 ± 0.10	0.71	0.05 ± 0.07	0.47
MIBK ^4^^)^	0.39 ± 0.46	3.09	0.37 ± 0.48	3.88	0.07 ± 0.07	0.39	0.07 ± 0.10	0.84
Toluene	2.98 ± 2.02	10.30	3.02 ± 2.77	15.39	1.14 ± 0.97	5.68	1.11 ± 1.18	8.81
Tetrachloroethylene	0.01 ± 0.03	0.11	0.03 ± 0.05	0.31	0.01 ± 0.01	0.07	0.01 ± 0.03	0.15
Ethylbenzene	0.33 ± 0.30	1.94	0.36 ± 0.35	1.97	0.15 ± 0.19	2.13	0.14 ± 0.11	0.81
*m,p*-Xylenes	0.56 ± 0.66	6.51	0.51 ± 0.46	2.44	0.23 ± 0.31	2.62	0.20 ± 0.15	1.02
Styrene	0.08 ± 0.09	0.57	0.07 ± 0.09	0.61	0.01 ± 0.02	0.10	0.02 ± 0.02	0.11
*o*-Xylene	0.18 ± 0.23	2.26	0.19 ± 0.19	1.33	0.08 ± 0.12	0.96	0.07 ± 0.05	0.34
1,2,4-TMB ^5^^)^	0.08 ± 0.16	1.76	0.07 ± 0.07	0.43	0.06 ± 0.13	1.00	0.04 ± 0.03	0.13
*N*,*N*-DMF ^6^^)^	0.16 ± 0.28	2.00	0.34 ± 0.58	3.31	0.04 ± 0.10	0.51	0.03 ± 0.08	0.70
Naphthalene	0.03 ± 0.03	0.15	0.03 ± 0.02	0.10	0.03 ± 0.02	0.11	0.03 ± 0.02	0.09
Formaldehyde *	3.77 ± 1.85	9.74	3.67 ± 1.68	7.59	3.17 ± 1.55	7.27	3.20 ± 1.63	8.41
Acetaldehyde *	2.58 ± 1.65	7.49	3.32 ± 1.70	8.05	1.43 ± 1.32	5.20	1.51 ± 1.22	6.00
Acetone *	0.49 ± 0.45	2.82	0.50 ± 0.65	3.12	0.35 ± 0.30	1.29	0.36 ± 0.31	3.42
MEK * ^7^^)^	1.05 ± 1.55	4.30	0.93 ± 0.85	4.73	0.55 ± 0.32	1.80	0.48 ± 0.40	2.13
Propionaldehyde *	0.02 ± 0.10	0.31	0.02 ± 0.12	0.31	0.02 ± 0.05	0.23	0.02 ± 0.04	0.25
Crotonaldehyde *	0.06 ± 0.15	0.66	0.07 ± 0.08	0.49	0.03 ± 0.05	0.22	0.02 ± 0.06	0.34

^1^^)^ Standard deviation; ^2^^)^ Maximum; ^3^^)^ Methy ter-butyl ether; ^4^^)^ Methyl iso-butyl ketone; ^5^^)^ 1,2,4-Trimethylbenzene; ^6^^)^ Dimethylformamide; ^7^^)^ Methyl ethyl ketone. * The number of samples for the carbonyl compounds is 53 for each site.

Although the overall rankings of individual compounds in each site were similar, the levels of some VOCs and carbonyls varied widely between the sampling sites, indicating the effects of local emission sources on the sites. For the first campaign, the mean concentrations of toluene, acetaldehyde, and trichloroethylene appeared to be much higher in industrial sites I and II than in the other sites (Table 3), whereas saturated hydrocarbons, such as pentane, decane and nonane, were detected not only more frequently but at higher levels in the industrial sampling site III. Interestingly, a large municipal landfill is located in the vicinity of this site. Thus, the increased levels of alkanes were estimated to be affected by the VOC emissions from the landfill [37,38].

For the second campaign, as shown in Table 4, there is also a clear tendency for some VOCs of higher levels at industrial sites than the other sites. According to the Korea PRTR Data [12], the major compounds used in industries located in Gumi City are toluene, ethylbenzene, *m,p*-xylenes, trichloroethylene, isopropyl alcohol, ethyl acetate, and styrene. All these VOCs were found generally at higher levels in industrial sites. On the other hand, the mean concentrations of benzene and methyl tert-butyl ether (MTBE) appeared to be similar at all four sites, suggesting that their major source is vehicle emissions. Although the annual average concentrations of formaldehyde for industrial site I and II were higher than that of the non-industrial sites, there was no statistically significant difference between the sites. This suggests that formaldehyde is emitted from a variety of sources, such as motor vehicles, furniture, insulation materials, and combustion, which are ubiquitous both indoors and outdoors in an urban area [7]. In addition, formaldehyde can be formed in the atmosphere as a byproduct of photochemical reactions [8,19]. DMF is a solvent used extensively in the textile industries for the dying process, and it was found to be higher at industrial site II than in the other sites because this site is surrounded by many textile industries. Although a few studies reported the DMF data in occupational settings [39], there is little information on the ambient levels for this compound.

### 3.3. Comparison of VOCs Concentrations in Industrial and Non-Industrial Areas

**Figure 2:**
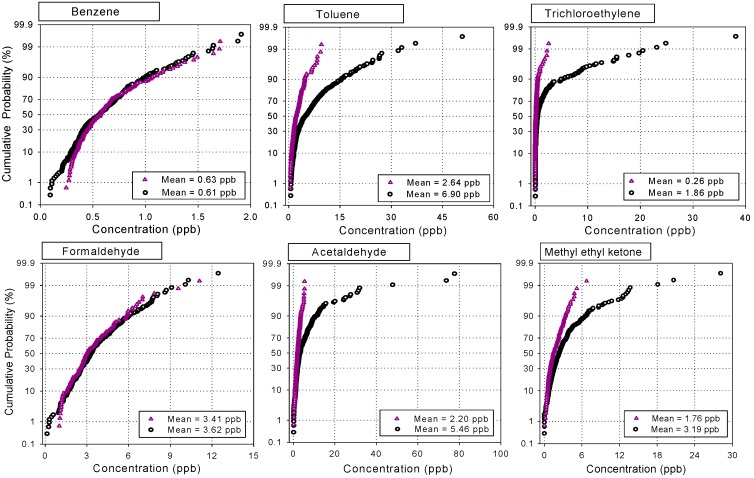
Comparison of the concentration distributions of selected VOCs in industrial (circle dot) and non-industrial areas (triangle dot) for the first campaign during 2003/2004.

**Figure 3:**
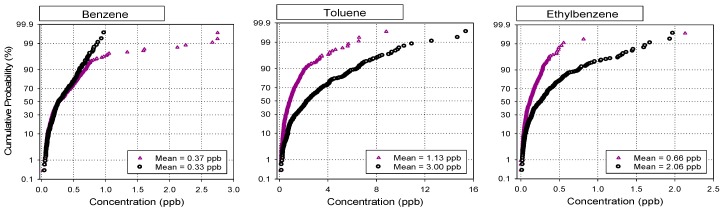

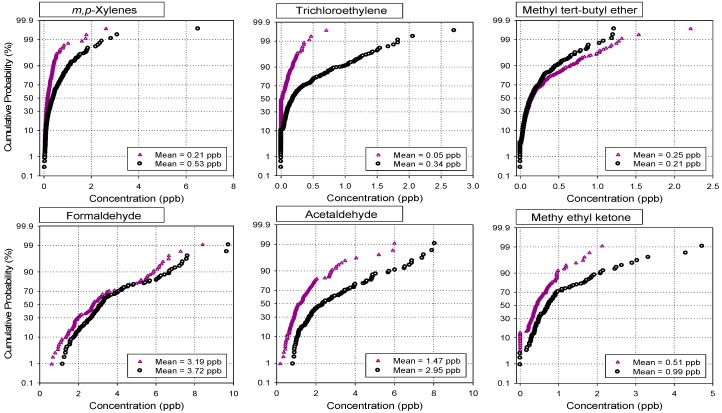
Comparison of the concentration distributions of selected VOCs in industrial (circle dot) and non-industrial areas (triangle dot) for the first campaign during 2010/2011.

To investigate the impacts of industrial sources on individual compounds in residential and commercial areas, the measured VOCs and carbonyl data were divided into two groups, *i.e.*, industrial and non-industrial groups. In other words, all the measured data from the industrial sites were pooled as a group, while the data from the residential and commercial sites was placed into another group. Comparisons of the two groups for the first and the second campaigns are illustrated in Figure 2 and Figure 3, respectively.

Figure 2 shows cumulative probabilities of the concentration data of selected VOCs for the two groups. Two distinct patterns can be found in Figure 2. Toluene, trichloroethylene, acetaldehyde, and MEK showed much higher levels in the industrial data group than the non-industrial group, whereas no statistically significant differences (α = 0.05) were found for benzene and formaldehyde between the two groups. This indicates that the major sources of VOCs with higher levels in Figure 2 were mostly industrial emissions. On the other hand, there was no evidence of potential impacts of industrial sources on the occurrence of benzene and formaldehyde in the ambient air of Gumi City.

As shown in Figure 3, the data from the second campaign also showed very similar patterns to the first campaign, even though the concentrations of the individual compounds are different from the previous survey. Distinct differences in the levels of toluene, ethylbenzene, xylenes, trichloroethylene, acetaldehyde, and MEK were noted between the two groups, indicating all these compounds are strongly associated with industrial emissions. The mean concentration of trichloroethylene in the industrial data group appeared to be almost 7 times higher than in the non-industrial group, whereas toluene, ethylbenzene and xylenes were 2~3 times higher. Acetaldehyde and MEK also showed approximately 2 times higher levels. In contrast, no significant differences in benzene and MTBE were noted between the two groups. As mentioned earlier, the Korean PRTR data also confirmed the non-use of benzene and MTBE in the industries in Gumi City [12]; thus the main source of benzene and MTBE in this city is believed to be vehicle emissions instead of industrial activities.

Acetaldehyde is a notorious mal-odorant but it has not been reported in the Korean PRTR, because this compound is not a common solvent or a raw material in the industry. Nevertheless, the high levels of acetaldehyde in Gumi City have been of concern for a long time due to the public complaints of foul smells; this issue was discussed in a previous paper [7]. Alcohols, such as methanol and isopropanol, can play a role as a precursor of acetaldehyde emitted from industrial processes [40,41]. In fact, it was reported that a large amount of those alcohols are being used in the electronic industries in Gumi industrial complexes [7,12].

### 3.4. Temporal Variations of VOCs Concentrations

**Figure 4:**
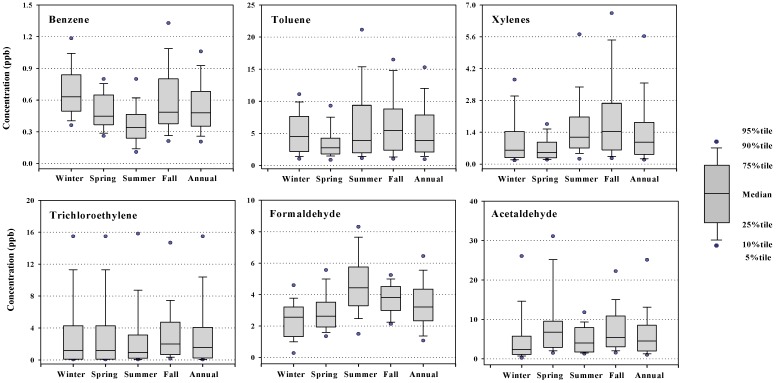
Seasonal variations of the selected VOCs at industrial site I during 2003/2004.

**Figure 5:**
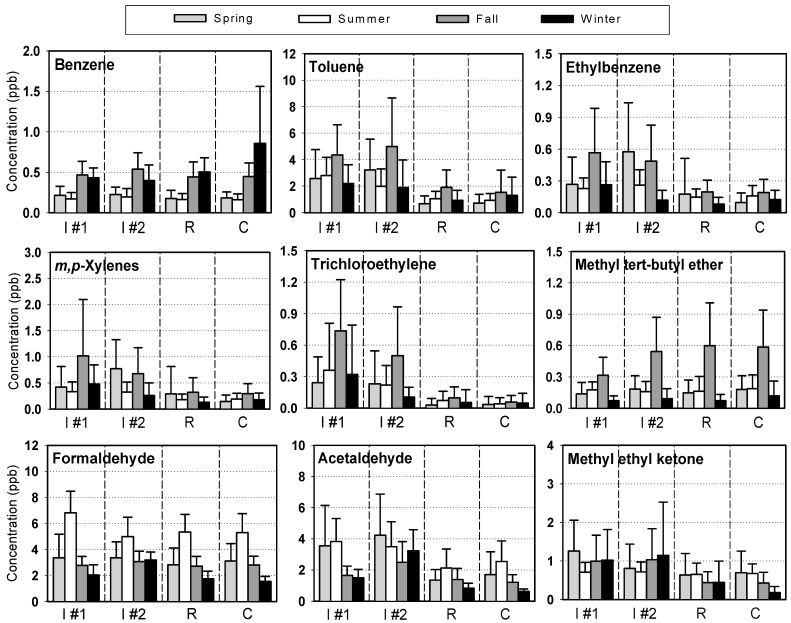
Seasonal variations of the selected VOCs at four different sites during 2010/2011 (the error bar indicates a standard deviation). I #1: industrial sit 1, I #2: industrial site #2, R: residential site, C: commercial site.

Seasonal variations in the ambient levels of VOCs can be influenced by a number of factors, including source variations, fuel consumption, chemical reactivity, meteorology, and the location and time of sampling. In the first campaign, seasonal variations of VOCs did not appear to be significantly different from each other. Therefore, seasonal data for industrial site I, which showed not only higher levels in most cases, but also more frequently measured than other sites, were demonstrated in Figure 4 for selected VOCs, as a typical example. The data from the second campaign were presented in Figure 5, where seasonal average concentrations of the selected VOCs in four sites were compared each other.

Examination of Figure 4 and Figure 5 showed that the seasonal patterns of benzene, formaldehyde, acetaldehyde and other VOCs were all different from each other. First of all, the benzene concentrations increased during the winter and fall seasons in both campaigns. In typical urban areas, where industrial emissions are ignored, increased levels of VOCs might be generally expected during the colder seasons than warmer seasons [8]. Although elevated temperatures in the summer will obviously increase the evaporation of VOCs, the decay or removal of VOCs through photochemical reactions will be more significant during the summer. In addition, the ambient VOCs levels tend to increase due to air stagnation during the cold season. VOCs emissions from cold start vehicles account for a large proportion of the total VOCs emissions from motor vehicles equipped with catalytic converters, particularly during the winter [8,38]. Therefore, the increased winter benzene levels observed in this study suggest that catalysts may not remove sufficient VOCs from the exhaust under stagnant winter conditions in Korea.

On the other hand, other VOCs associated with industrial emissions, such as toluene, ethylbenzene, xylenes and trichloroethylene, showed relatively fewer variations throughout the year, but were generally higher in fall than in the other seasons. Assuming that the industrial activity in Gumi City is constant throughout a year, the relatively higher concentrations during the fall season might be due to meteorological conditions. The meteorological conditions during both campaigns are summarized in Table 5. The data was obtained from the Gumi Regional Meteorological office, which is located near the commercial sampling site (within a distance of 500 m from the site C in Figure 1). Interestingly, the average wind speed for the sampling period for the fall season was 1.2 m/s for both campaigns, which was the lowest among the four seasons in 2010, and lower than winter and spring, but similar to the summer in 2004. No precipitation was noted during this period. Therefore, such elevated levels of industrial VOCs in the fall might be caused by the limited dilution, no scavenging, and poor dispersion in the atmosphere.

In contrast to benzene, the formaldehyde levels appeared to be the highest in summer and lowest in winter (Figure 4 and Figure 5), showing a typical “high-in-summer and low-in-winter” pattern, which has been reported elsewhere [35,36,42,43]. The increased concentrations of formaldehyde in summer were attributable not only to the increased volatile emissions due to the higher temperature, but also to the formation of secondary pollutants as a byproduct of the photochemical reactions in summer [40,43,44]. Acetaldehyde, methyl ethyl ketone and acetone are also known to be emitted as secondary pollutants of photochemical processes [45]. In this study, however, the concentrations of these carbonyl compounds did not vary noticeably from season to season, indicating that these compounds are largely associated with local emission sources with a variety of independent and irregular industrial activities.

**Table 5:** Meteorological conditions during the first and the second campaigns in Gumi City.

**2003/2004 Campaign**							
**Sampling Period**	**Season**	**Average temp. (°C)**		**Wind Speed (m/s)**	**Prevailing Wind Direction**		**Rain (mm)**
13~19 January	Winter	1.6		2.0	W		1.0
9~15 April	Spring	20.2		1.4	W		0.0
13~19 August	Summer	26.2		1.0	WSW		56.5
15~21 October	Fall	17.1		1.2	NW		0.0
**2010/2011 Campaign**							
**Sampling Period**	**Season**	**Average temp. (°C)**	**Wind Speed (m/s)**			**Prevailing Wind Direction**	**Rain (mm)**
21~27 May	Spring	17.8	1.4			WNW	50.5
3~9 August	Summer	28.3	1.5			WNW	57.0
15~21 October	Fall	14.3	1.2			WNW	0
5~11 January	Winter	−3.0	2.8			WNW	0

**Figure 6:**
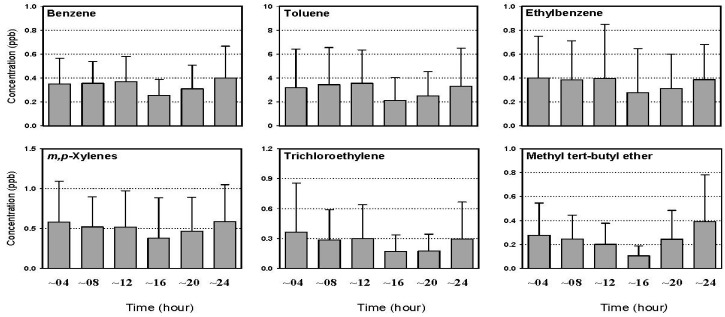
Diurnal variations of the 4 hourly average concentrations of selected VOCs at industrial site II during the 2010/2011 campaign (the error bar indicates a standard deviation).

During the first campaign, to investigate diurnal variations in the VOCs levels, the samples were taken three times per day, *i.e.*, in the morning, afternoon and evening. More detailed diurnal variations were investigated during the second campaign by collecting six samples consecutively in a day. Figure 6 gives an example of the daily variations of the VOCs measured at industrial site II. This site was selected as an example because it is located in a mixed zone of industrial, residential and commercial areas. The concentrations of VOCs during a day varied, depending on the sites and compounds, but it was generally noticed that the levels of most VOCs, except formaldehyde, increased in the morning, decreased in the afternoon, and then increased again in the evening. The rise and fall in the VOCs levels during a day is apparently due not only to the atmospheric stability but also to traffic volumes in the Gumi area. In general, the traffic volume increases in the morning and evening. In contrast, the mixing height increases in the afternoon, but decreases in the morning and evening. In most cases, however, the concentrations of formaldehyde increased during the afternoon, particularly in summer. This suggests that there is a contributive portion of the secondary formation of formaldehyde through photochemical reactions in the air, as mentioned previously in this paper.

### 3.5. Comparison of Concentrations of VOCs in 2003–2004 and 2010–2011 in Gumi City

An assessment of spatial and temporal variations of the VOCs concentrations in Gumi City suggests that there might have been significant changes in air quality in the city during the 7 year period. To evaluate the long term trends in the levels of VOCs and carbonyls in the city, site-by-site comparisons were carried out for commonly measured compounds in both campaigns, and the results are presented in Table 6. For comparison, the % reduction was calculated for each compound of concern from the data in Table 3 and Table 4, which is defined as a percentage of the difference between the two campaigns relative to the data from the first campaign. Table 6 shows that in most cases, the concentrations of VOCs and carbonyls have decreased considerably in the second campaign compared to the first campaign. The only exception is formaldehyde, which showed no significant reductions during the seven year period. Other VOCs and carbonyls appeared to be reduced in the range of 21.3%~98.2%, depending on the sampling sites. The largest reduction was found for dichloromethane, which was 93.6% on city-wide average, whereas other chlorinated VOCs, such as trichloroethylene and tetrachloroethylene, were reduced to approximately 25% of that measured in the first campaign. Aromatic VOCs, such as toluene, xylenes and ethylbenzene, also showed 43.0%~55.4% reductions, indicating that the concentrations of these compounds decreased to almost half of those measured in the first campaign.

**Table 6:** Estimation of the reductions of the average concentrations (in ppb) of selected VOCs in the 2010/2011 campaign relative to the 2003/2004 campaign in Gumi city.

	% Reduction by Mean Concentrations in 2010/2011 Relative to 2003/2004				
	Industrial Site I	Industrial Site II	Residential Site	Commercial Site	Total Mean ± SD
Dichloromethane	97.6	85.7	98.2	93.1	93.6 ± 5.8
*n*-Hexane	56.6	50.9	27.7	38.5	43.4 ± 15.5
Benzene	40.7	50.0	46.7	38.8	44.1 ± 5.2
Trichloroethylene	86.9	44.9	70.0	83.9	71.4 ± 19.1
Toluene	45.8	61.9	59.6	54.3	55.4 ± 7.1
Tetrachloroethylene	92.3	62.5	75.0	80.0	77.5 ± 12.3
Ethylbenzene	38.9	33.3	46.4	58.8	44.4 ± 11.0
*m,p*-Xylenes	49.1	46.9	59.6	58.3	53.5 ± 6.4
Styrene	65.2	30.0	80.0	60.0	58.8 ± 21.0
*o*-Xylene	40.0	32.1	50.0	50.0	43.0 ± 8.7
1,2,4-Trimethylbenzene	55.6	65.0	45.5	69.2	58.8 ± 10.6
Formaldehyde	−10.9	4.9	8.4	4.8	1.8 ± 8.6
Acetaldehyde	64.9	21.3	38.9	26.3	37.9 ± 19.5
Acetone	85.1	86.1	87.2	86.6	86.2 ± 0.9
Methyl ethyl ketone	56.3	72.0	69.4	71.9	67.4 ± 7.5

The decreased trend for the organic solvents was confirmed further using the Korea National PRTR database [12]. According to the PRTR data, 305 tons of toluene were emitted into the Gumi atmosphere during 2004, whereas 106 tons were released in 2010, showing a 65.2% reduction. This study showed that the ambient concentrations of toluene decreased by 45.8% and 61.9% in the industrial sites I and II, respectively. A similar trend was also observed in the case of xylenes (as a sum of *m*-, *p*- and *o*-), *i.e.*, a 44.0% reduction in the emission data and a 48.3% reduction in the ambient concentrations. In case of trichloroethylene, however, the present study found a greater reduction (65.9% as an average of two industrial sites) than the PRTR database, which is 52.6%. Confirmation of the reduced emissions for the other compounds is not possible due to a lack of information in the 2004 PRTR database. Nevertheless, this study clearly shows that a reduction of the industrial emissions of VOCs and other organic solvents, such as acetone and MEK, resulted in decreased concentrations in the ambient atmosphere of the industrial areas, which led to decreased levels of those compounds in the air of the residential and commercial areas in Gumi City.

Very high levels of acetaldehyde in industrial site I (Table 3) have been of concern in Gumi City, and a previous study [7] suggested the necessity of intensive supervision and surveillance for this compound as one of the top priority pollutants in the city. Interestingly, it was reported that the number of public complaints of foul smells in Gumi City had reduced considerably in 2009 compared to previous years [46], which was attributed to the decreased concentrations of acetaldehyde in industrial areas, as shown in Table 6. The mean concentration of benzene, a group 1 carcinogen, was reduced by approximately 44.1% on average in the four sites.

The considerable reductions in the concentrations of VOCs during a period between the two campaigns can be attributed to many reasons. First of all, governmental regulations for VOCs and HAPs have been strengthened during this period [1]. In Korea, the number of HAPs was 25 until 2007, but 9 VOCs and a group of polycyclic aromatic hydrocarbons (PAHs) were added to the list in 2008. Among the 35 HAPs, 24 chemicals (or a class of chemicals) are organic compounds. The emission standards for HAPs have been strengthened steadily over the last 20 years. According to the Korean air quality preservation law, if an industry deals with chemicals included in the HAPs list, very strict obligations are applied to the company for permission, monitoring, reporting, installation and operation of control facilities, *etc*. In addition to governmental policies, voluntary agreements between major companies and the Ministry of Environment to reduce the use of organic solvents appear to play a contributory role in reducing VOC emissions [1]. The introduction of advanced technology to control VOCs emissions in major industries also appear to contribute to the decrease in the concentrations of atmospheric VOCs in the city. Finally, the improvement in air quality is likely to be associated with the incidental effects of a site planning project implemented by Gumi city government. The project has been started in 2005 and is still on-going to redevelop the old industrial complex as an “eco-friendly” complex. Many old companies have been moved to a new industrial complex in the outskirts of the city (approximately 10 to 20 km away in northeast direction from the old complexes), and the empty spaces are being developed as a neighborhood park [4].

## 4. Conclusions

This paper reported the results of two field monitoring campaigns at a seven year interval in Gumi City, which was developed specifically for the electronic industry in Korea in the 1970s. More than 80 individual compounds were measured in this study, and then important compounds were identified with respect to their concentrations, frequencies of detection, and toxicities. The monitoring data showed that toluene, trichloroethylene and acetaldehyde are the most significant toxic VOCs in Gumi City, and their major sources are mainly industrial activities. On the other hand, there was no clear evidence of the industrial impacts on the concentrations of benzene and formaldehyde in the ambient air of the city. The ambient concentrations of benzene appeared to be similar at all sampling sites, suggesting that its major source is vehicle emissions. Formaldehyde also showed a similar pattern to benzene, but an additional contribution of secondary formation in the atmosphere to the ambient levels was observed in the summertime. Overall, the seasonal variations were not as distinct as locational variations in the VOCs concentrations, whereas the diurnal variations showed a typical pattern of urban air pollution, *i.e.*, increase in the morning, decrease in the afternoon and an increase again in the evening.

In most cases, there were considerable reductions in the VOCs concentrations from 2004 to 2011 in Gumi City. Citywide average reductions of 55.4%, 48.3% and 71.4% for the concentrations of toluene, xylenes and trichloroethylene, respectively, were estimated. The reductions in the ambient concentrations for the three compounds were confirmed by the Korean PRTR data for industrial emissions within the city during the same period. Significant reductions in concentrations of benzene and acetaldehyde were also observed, whereas formaldehyde appeared to be relatively constant during the period. The decreased trends in the concentrations of VOCs and carbonyls were attributed not only to the stricter regulations on VOCs in Korea, but also to the voluntary cooperation and introduction of advanced technology to control VOCs emissions in major industries. In addition, a site planning project by Gumi City for redeveloping the old industrial complex as an eco-friendly complex is likely to play a contributory role in improving the air quality of the city.
